# ING2 (inhibitor of growth protein-2) plays a crucial role in preimplantation development

**DOI:** 10.1017/S0967199414000768

**Published:** 2015-02-12

**Authors:** Lin Zhou, Pei Wang, Juanjuan Zhang, Boon Chin Heng, Guo Qing Tong

**Affiliations:** 1 Reproduction Medicine Center, Shuguang Hospital Affiliated to Shanghai University of Traditional Chinese Medicine, 528 Zhangheng Rd, Shanghai 201203, PR China.; 2 State Key Laboratory of Reproductive Medicine, Department of Reproduction, Nanjing Maternity and Child Health Care Hospital Affiliated to Nanjing Medical University, Nanjing 210004, PR China.; 3 Sunway University, Department of Biological Sciences, Faculty of Science & Technology, 5 Jalan Universiti, Bandar Sunway 47500, Selangor Darul Ehsan, Malaysia.; 4 Department of Biosystems Science & Engineering (D-BSSE), ETH-Zurich, Mattenstrasse 26, Basel 4058, Switzerland.; 5 Reproduction Medicine Center, Shuguang Hospital Affiliated to Shanghai University of Traditional Chinese Medicine, 528, Zhangheng Rd, Shanghai 201203, PR China.

**Keywords:** ING2, Inhibitor of growth protein, Preimplantation development

## Abstract

ING2 (inhibitor of growth protein-2) is a member of the ING-gene family and participates in diverse cellular processes involving tumor suppression, DNA repair, cell cycle regulation, and cellular senescence. As a subunit of the Sin3 histone deacetylase complex co-repressor complex, ING2 binds to H3K4me3 to regulate chromatin modification and gene expression. Additionally, ING2 recruits histone methyltransferase (HMT) activity for gene repression, which is independent of the HDAC class I or II pathway. However, the physiological function of ING2 in mouse preimplantation embryo development has not yet been characterized previously. The expression, localization and function of ING2 during preimplantation development were investigated in this study. We showed increasing expression of ING2 within the nucleus from the 4-cell embryo stage onwards; and that down-regulation of ING2 expression by endoribonuclease-prepared small interfering RNA (esiRNA) microinjection results in developmental arrest during the morula to blastocyst transition. Embryonic cells microinjected with ING2-specific esiRNA exhibited decreased blastulation rate compared to the negative control. Further investigation of the underlying mechanism indicated that down-regulation of ING2 significantly increased expression of *p21*, whilst decreasing expression of *HDAC1.* These results suggest that ING2 may play a crucial role in the process of preimplantation embryo development through chromatin regulation.

## Introduction

Preimplantation embryo development includes fertilization, cell cleavage, morula and blastocyst formation. The formation of a healthy blastocyst is critical for subsequent implantation, pregnancy and fetal development (Watson & Barcroft, 2001). The preimplantation embryo exhibits an autonomous development capability influenced by various factors within the maternal and embryonic environment (Schultz, 2005). Similarly, *in vitro* culture conditions also exert crucial effects on embryo developmental potential (Niemann & Wrenzycki, 2000; Schultz & Williams, 2002; Ecker *et al.*, 2004). In clinical assisted reproductive technology, embryos are selected for transfer based mainly on morphological criteria, which to some extent reflect their developmental potential. In most cases, day-2 or day-3 cleavage-stage embryos are usually transferred. Human embryonic genome activation (EGA) occurs from the 4–8-cell stage (Braude *et al.*, 1988), which makes embryo selection at earlier developmental stages less reliable. In reality, prolonged culture is not always utilized as a selection tool because aneuploidies or aberrant gene expression prevent development to the blastocyst stage in a large proportion of embryos (Gardner *et al.*, 1998; Dal Canto *et al.*, 2012). Hence, it is imperative to characterize the mechanisms controlling preimplantation development, to further our understanding of basic reproductive biology, as well as advance practical applications in the clinic.

Preimplantation embryonic development is regulated both genetically and epigenetically. The global gene expression profile is dynamic and displays stage-specific characteristics at different developmental stages of the preimplantation embryo. For example, Oct4, Nanog, Gata3 and Bmp8b are activated between the 4–8-cell embryonic stage (Hamatani *et al.*, 2004). These genes are key regulators of differentiation into the inner cell mass (ICM) and trophectoderm (TE), which is the very first cell lineage specification in mouse embryonic development (Yamanaka *et al.*, 2006). Many studies have suggested that DNA methylation and the regulation of histone modifications have profound effects on preimplantation embryonic development (Monk *et al.*, 1987; Santos *et al.*, 2005; Yeo *et al.*, 2005; Torres-Padilla *et al.*, 2006). Two major histone deacetylase (HDAC) complexes (NuRD and SIN3) have specific functions in embryonic development (Ahringer, 2000). HDAC1 is a major deacetylase in preimplantation embryos and its expression negatively regulates the acetylation state of histone H4K5 during preimplantation embryonic development (Ma & Schultz, 2008). Although knowledge of the mechanisms of preimplantation development has increased gradually over the past decade, the key transcription factors that are essential for blastocyst formation and how they interact with epigenetic regulators have yet to be characterized.

ING2 participates in diverse cellular processes involving tumor suppression, DNA repair, cell cycle regulation, and cellular senescence, all of which are functionally linked to the p53 tumor suppressor protein (Unoki *et al.*, 2008; Zhang *et al.*, 2008; Larrieu *et al.*, 2009). Additionally, ING2 has biological functions that are independent of the p53 pathway. As a basic subunit of the histone deacetylase Sin3 complex, ING2 mediates the binding of the Sin3 complex to some promoters where di-/tri-methylated H3K4 regulates gene repression (Smith *et al.*, 2010). ING2 can also play a role in modulating histone methyltransferase (HMT) activity associated with silencing function, which involves a non-HDAC class I or II pathway (Goeman *et al.*, 2008). As ING2 modulates both gene expression and chromatin modification, this study attempted to examine the role of ING2 in mouse preimplantation embryonic development.

## Materials and methods

All chemicals and culture media were purchased from Sigma-Aldrich Inc. (St. Louis, MO, USA), unless stated otherwise.

### Embryo collection and culture

The ICR mice were fed *ad libitum* with a standard diet and were housed in a room with controlled temperature and lighting (20–22°C, 12 h/12 h light/dark cycle), in accordance with the Institutional Animal Care and Use Committee (IACUC) guidelines of Nanjing Medical University. For the collection of zygotes, 8-week-old female ICR mice were injected with human chorionic gonadotropin (HCG) and mated with male ICR mice shortly after injection. After 21 h, zygotes were collected from the oviducts of the mated female mice and transferred into 4-(2-hydroxyethyl)-1-piperazineethanesulfonic acid (HEPES)-buffered Chatot, Ziomet and Bavister (CZB) medium. Cumulus cells were dispersed by treatment with 1 mg/ml hyaluronidase in HEPES-CZB. Cumulus-free zygotes were washed with HEPES-CZB medium and then cultured in CZB medium until they developed to later cleavage-stage embryos. All cultures were carried out within a humidified 5% CO_2_ incubator at 37°C.

### Immunofluorescence and confocal microscopy

For immunofluorescence staining of ING2, the embryos were fixed in 4% paraformaldehyde in phosphate-buffered saline (PBS; pH 7.4) for at least 30 min at room temperature. After being permeabilized with 0.5% Triton X-100 at room temperature for 20 min, the embryos were incubated for 1 h in blocking solution composed of 1% bovine serum albumin in PBS, followed by incubation with goat anti-ING2 antibody (dilution, 1:100) overnight at 4°C. After three washes in PBS containing 0.1% Tween 20 and 0.01% Triton X-100 for 5 min each, the embryos were labelled with fluorescein isothiocyanate-conjugated rabbit anti-goat IgG (dilution, 1:100) for 1 h at room temperature. After three washes in PBS containing 0.1% Tween 20 and 0.01% Triton X-100, the embryos were co-stained with propidium iodide (10 μg/ml in PBS), prior to being mounted on glass slides and examined under a confocal laser scanning microscope (Zeiss LSM 510 META, Jena Germany).

### Immunoblotting analysis

The extracted proteins of adult ICR mouse ovaries and morphologically normal 4-cell embryos (150 embryos/sample) were separated by sodium dodecyl sulfate polyacrylamide gel electrophoresis (SDS-PAGE) and then subsequently transferred to polyvinylidene fluoride membranes via electrophoresis. Following the transfer, the membranes were blocked in TBST (TBS buffer with 0.1% Tween 20) containing 5% skimmed milk for 2 h, followed by incubation overnight at 4°C with goat polyclonal anti-ING2 antibody (dilution, 1:500; sc-67646, Santa Cruz, Dallas, TX, USA). After being washed in TBST three times for 10 min each, the membranes were incubated for 1 h at 37°C with horseradish peroxidase-conjugated rabbit anti-goat IgG (dilution, 1:1000). Finally, the membranes were processed and analyzed using an enhanced chemiluminescence detection system (Amersham, Piscataway, NJ, USA).

### Reverse transcription polymerase chain reaction (RT-PCR)

The primer sequences utilized for RT-PCR analysis of *Ing2* expression were as follows: sense, 5′-GGGAGCTGGACAACACCTACCAA-3′ and antisense, 5′-TTCGAGCATCTGGGTGACAATC-3′. Total RNA extraction and RT were performed using an RNeasy Micro Kit (Qiagen, Valencia, CA, USA), according to the manufacturer's instructions; with oligo-dT being utilized as a primer. Real-time PCR analysis was carried out using a Faststart Universal SYBR Green Master Mix (no. 04913914001, Roche Inc., Basel, Switzerland), with an ABI Prism 7500 System (Applied Biosystems, Foster City, CA, USA). We processed 15–30 embryos at a time, and the *H3f3a* gene was utilized as an endogenous control (May *et al.*, 2009). Relative quantitation of target gene expression was evaluated by the 2^−ΔΔCt^ method (Livak & Schmittgen, 2001) and data were collated from three replicate experiments utilizing different sets of embryos.

### ING2 MISSION® esiRNA and *in vitro* culture

ING2 MISSION® esiRNA (EMU022101) and negative control EGFP MISSION® esiRNA (EHUEGFP) were purchased from Sigma-Aldrich Inc. (St. Louis, MO, USA), which possessed the intellectual property to design and prepare the MISSION esiRNA. The *Ing2* cDNA target sequence is as follows: 5′-AAACGCCTACAGCAGCATCTCCAGAGAGCGTTAATCAATAGCCAAGAATTGGGAGATGAAAAAATTCAGATTGTCACCCAGATGCTCGAATTGGTGGAGAACCGAGCGAGACAAATGGAGCTGCATTCACAGTGTTTCCAGGATCCTGCTGAAAGTGAGCGAGCCTCAGACAAGTCGAAGATGGATTCCAGTCAACCGGAAAGATCTTCTAGAAGACCTCGAAGACAGAGGACCAGTGAGAGCCGTGACTTATGTCACATGACAAACGGGATTGACGACTGTGATGATCAACCACCGAAAGAAAAGAGATCCAAGTCCGCCAAGAAGAAGAAGCGCTCCAAGGCCAAGCAGGAGAGGGAGGCATCCCCTGTCGAGTTTGCCATCGATCCCAATGAGCCCACCTACTGCTTGTGTAACCAAGTGTCCTACGGGGAGATGATAGGCTG-3′. MISSION esiRNA are a heterogeneous mixture of siRNAs that all target the same mRNA sequence. These multiple silencing triggers lead to lower off-target effects than single or pooled siRNAs, and exert highly specific and effective gene silencing. The concentration of ING2 MISSION esiRNA was 200 ng/μl in nuclease-free TE buffer (10 mM Tris-HCl, pH 8.0, 1 mM EDTA). Approximately 10 pl of individual esiRNA was microinjected into the cytoplasm of the zygotes, which were cultured in CZB medium and subjected to further observation. The developmental progress of each group was observed and analyzed using a Nikon TE2000-S microscope.

### Statistical analysis

Each experiment was repeated at least three times. Before statistical analysis, all percentage data were subjected to arc-sine transformation. Data were analyzed with one-way analysis of variance, paired Student's *t*-test and the chi-squared test, using SPSS software (SPSS Inc., Chicago, IL). Data are expressed as mean ± standard deviation (SD), with a *P*-value < 0.05 being considered statistically significant.

## Results

### The expression of ING2 during mouse preimplantation development

**Figure 1:**
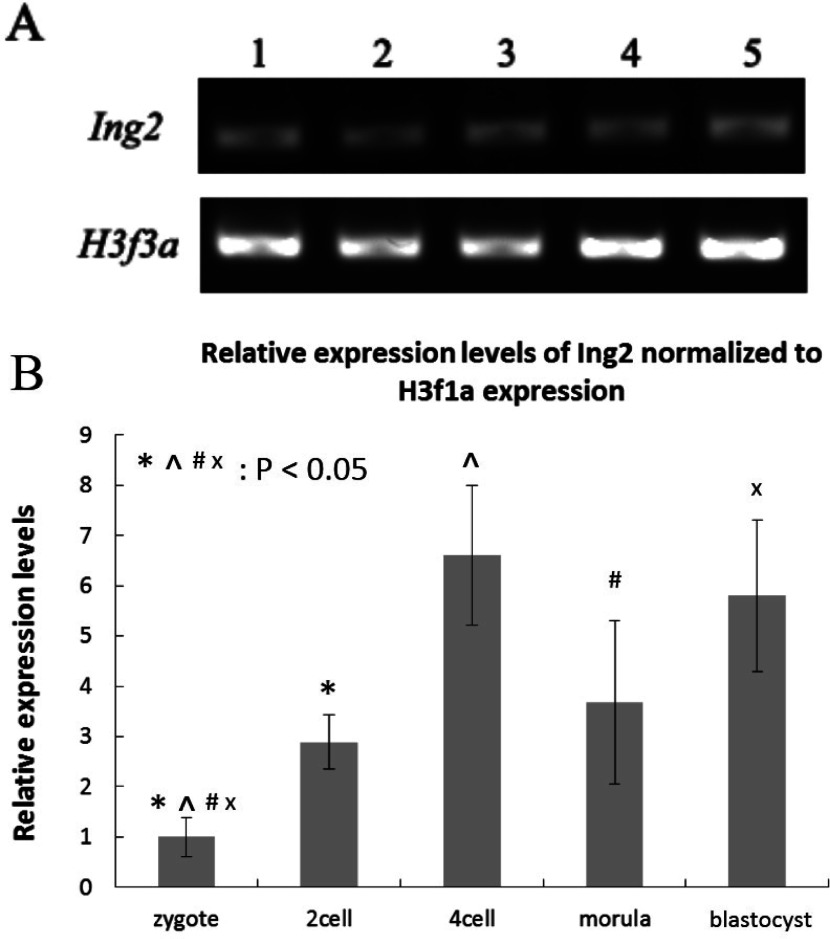
(*A*) SDS-PAGE gel of RT-PCR amplification of *Ing2* mRNA transcripts extracted at different embryonic stages. *H3f3a* was utilized as the endogenous reference gene. Lanes 1, 2, 3, 4, 5 corresponds to zygote, 2-cell embryo, 4-cell embryo, morula-stage embryo and blastocyst-stage embryo respectively. (*B*) Real-time PCR analysis of *Ing2* mRNA expression patterns at different embryonic stages during mouse preimplantation development. The expression level was calculated from the cycle threshold (C_t_) values by the 2^−ΔΔCt^ method. The calibration sample was embryos at the zygote stage. Bar graphs indicate mean ± standard deviation (SD) of three experimental replicates.

**Figure 2:**
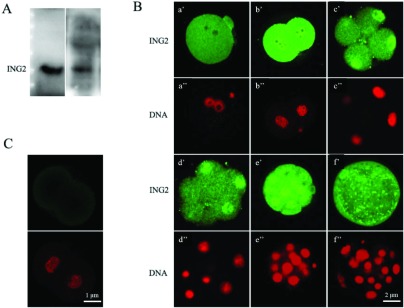
(*A*) Western blot with the anti–ING2 antibody showed only one distinct band at the predicted molecular weight of ING2 protein (33 kD), for both the mouse ovary and 4-cell embryo. (*B*) Immunofluorescence staining of ING2 and nuclear DNA in preimplantation embryos with specific anti-ING2 antibody (green, *a′–f′*) and propidium iodide (red, *a′′–f′′*) respectively. zygote, approximately 21 h after human chorionic gonadotropin (HCG) injection (*a′*, *a′′*); 2-cell embryo, approximately 45 h after HCG injection (*b′*, *b′′*); 4- to 8-cell embryo, approximately 58 h after HCG injection (*c′*, *c′′*, *d′*, *d′′*); morula, approximately 78 h after HCG injection (e,′ e′′) and blastocyst, approximately 96 h after HCG injection (*f′*, *f′′*). Scale bar: 2 μm. (*C*) Unstained 2-cell embryo was utilized as the negative control. Scale bar: 1 μm.

We analyzed the expression profile of *Ing2* at each stage of mouse preimplantation embryonic development. The RT-PCR data confirmed expression of *Ing2* in mouse preimplantation embryos (Fig. 1*A*), and further analysis revealed variation in expression levels of *Ing2* at different embryonic developmental stages (Fig. 1*B*). Most notably, *Ing2* expression rapidly increased from the 2-cell to 4-cell cleavage-stage. This suggests that *Ing2* may be a crucial modulator of early embryonic development genes. Western blot analysis utilizing an anti-ING2 antibody revealed an exclusive band at the expected molecular mass of 33 kDa, for both the mouse ovary and 4-cell embryo (Fig. 2*A*).

### The localization of ING2 during mouse preimplantation development

Laser scanning confocal microscope was used to analyze subcellular localization of ING2 protein at different stages of the mouse preimplantation embryo. There was increased expression of ING2 from the 2-cell embryo stage onwards with increasing accumulation within the nucleus beginning from the 4-cell embryo stage (Fig. 2*B*). Unstained 2-cell embryos were utilized as the negative controls (Fig. 2*C*). The immunostaining result is consistent with the mRNA expression patterns revealed by RT-PCR. ING2 C-terminal contains a nuclear localization sequence (NLS) domain, which possesses three nuclear targeting sequences (NTS) that target to the nucleolus. Hence, the nuclear localization of ING2 thus suggests that ING2 may have key functions in regulating gene transcription or chromosome modification.

### Knockdown of ING2 retards embryonic development *in vitro*

**Figure 3:**
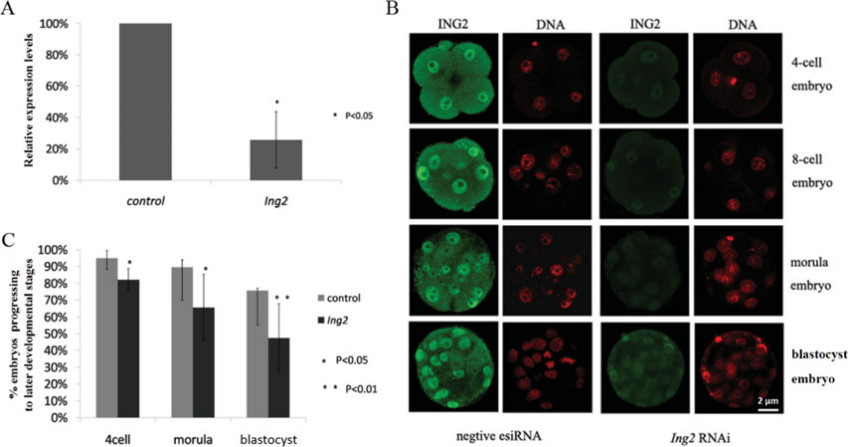
Zygotes were microinjected with ING2 specific sigma MISSION esiRNA. (*A*) Real-time PCR detected down-regulation of *Ing2* expression. The control and calibration sample was untreated zygotes. Error bars represent standard deviation (SD) (*P < 0.05). (*B*) Immunofluorescence staining confirmed decreased ING2 protein expression levels in the microinjected mouse embryos. (*C*) After zygotes were microinjected with ING2 specific esiRNA, the ratio of embryos that progressed to later embryonic developmental stages was evaluated. The depletion of ING2 led to developmental retardation. The results of five independent experiments were collated (*P < 0.05, **P < 0.01).

To investigate the role of ING2 in mouse embryos, zygotes were microinjected with sigma MISSION esiRNA against ING2. Zygotes microinjected with sigma negative MISSION esiRNA served as controls. Real-time PCR showed that the expression level of ING2 was significantly reduced (Fig. 3*A*) upon microinjection with the esiRNA, and this was further confirmed by reduced expression at the protein level detected by immunostaining (Fig. 3*B*). Inverted light microscopy was used to track the development of the treated embryos that were cultured up to the blastocyst stage (Fig. 3*C*). Notably, statistical analysis showed significant differences in the ratio of embryos that developed to the 4-cell, morula and blastocyst stage between the treated group (*n* = 150) and the negative control group (*n* = 132): 82.7% vs. 94.7% (*P* < 0.05), 67.3% vs. 89.4% (*P* < 0.05), 48.7% vs. 75.8% (*P* < 0.01) (Table 1). This demonstrated that ING2 expression was essential to mouse preimplantation embryonic development.

**Table 1:** The developmental competence of embryos that were microinjected with ING2-specific esiRNA or negative esiRNA was evaluated by determining the ratios of microinjected embryos progressing to various developmental stages

	No. of embryos (%)			
Treatment	Total	4-cell	Morula	Blastocyst
Ing2-esiRNA	150	124 (82.7)	101 (67.3)	73 (48.7)
Control	132	125 (94.7)**	118 (89.4)**	100 (75.8)**

** Values were considered significantly different from the control at *P* < 0.01 (vs. control).

### RNAi-mediated ablation of *Ing2* results in increased *p21* and decreased *HDAC1* expression

**Figure 4:**
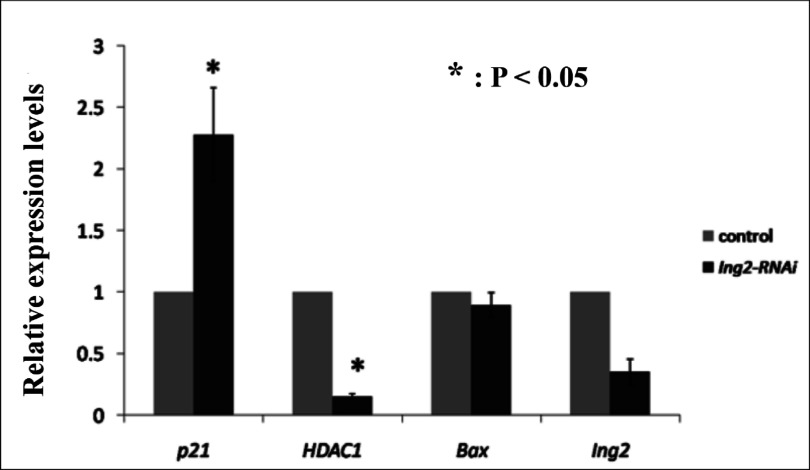
Real-time PCR analysis of expression of *Ing2*-associated genes in early blastocyst stage embryos after microinjection with ING2 specific esiRNA. The control and calibration sample was untreated embryos at the same developmental stage (early blastocyst). Data are presented as the mean of at least three biological replicates. Bar graphs indicate mean ± standard deviation (SD); *P < 0.05 vs. control.

To gain a better understanding of the basis for the developmental delay of the *Ing2*-depleted embryos, we assayed expression of *p21, Bax*, and *HDAC1* in these embryos by real-time PCR. The rationale for doing so is that ING2 activation leads to up-regulation of *p21* and *Bax* in a number of cancer cell lines (Nagashima *et al.*, 2001). Additionally, HDAC inhibitors have been shown to induce expression of *p21* in many cell types and tumors, leading to cell cycle arrest (Zhu *et al.*, 2004). Induction of *p21* was also observed in Hdac1-deficient embryonic stem cells (Lagger *et al.*, 2002). In this study, we observed a 2.3-fold increase in *p21* mRNA expression level at the early blastocyst stage (96 h post-hCG injection), while there was a corresponding 85% decrease in *HDAC1* mRNA level at the same time point in *Ing2*-depleted embryos (Fig. 4). It is hypothesized that the ING2-induced developmental delay could be due to decreased expression of *HDAC1* and concomitant increased expression of *p21*, and may not be associated with apoptosis because there was no increase in the expression of the pro-apoptotic *Bax* transcripts (Fig. 4).

## Discussion

In this study, we investigated the expression and localization of ING2 during mouse preimplantation development. We also demonstrated its physiological function in embryonic development and made preliminary investigations on its mechanism of action.

RT-PCR analysis revealed that there was a marked increase in *Ing2* expression from the 2-cell to 4-cell embryo stage. This is consistent with the immunostaining results, which showed an increase in expression of ING2 at the protein level from the 2-cell embryo stage, together with increasing localization within the nucleus of 4-cell blastomeres. In mouse, the first upsurge of gene expression occurs at the 2-cell embryo stage, and is known as zygotic genome activation (ZGA) (Flach *et al.*, 1982; Latham *et al.*, 1992; Nothias *et al.*, 1995). From then onwards, the embryo will exploit its own transcriptional and translational machinery to carry out its developmental program. This enables reprogramming of the newly formed embryonic genome to a totipotent state, which is subsequently lost at the blastocyst stage (Duranthon *et al.*, 2008; Calle *et al.*, 2012). Previous research suggested that genes activated from the transition of fertilized eggs to 4-cell embryos contribute mainly to the regulation of basic cellular machinery, while genes activated from transition of 8-cell embryos to the morula and blastocyst stages are mainly associated with dramatic biological and morphological events (Hamatani *et al.*, 2004). The spatio-temporal expression pattern of ING2, may give some cues on how it may contribute to mouse preimplantation development. Further investigation showed that down-regulation of ING2 by RNAi led to a decreased blastocyst formation rate compared with the negative control group, which means that ING2 does play a crucial role in preimplantation development.

As a growth inhibition factor, ING2 has a PHD domain that enables its interaction with H3K4Me3 to regulate gene repression (Gozani *et al.*, 2003; Shi *et al.*, 2006). ING2 is a stable component of the Sin3–HDAC complex. It has been shown that ING2 negatively regulates cell proliferation, chromatin remodeling, apoptosis and DNA repair through modulation of the p53 acetylation pathway, which incidentally enhance its own transcription (Wang *et al.*, 2006; Unoki *et al.*, 2008). Additionally, ING2 has also been implicated in muscle differentiation through regulation of myogenin transcription (Eapen *et al.*, 2012). The tri-methylated histone mark is found mainly in transcriptionally active regions (Bannister & Kouzarides, 2004). ING2, being a stable component of the Sin3–HDAC complex, recruits and stabilizes the Sin3–HDAC complex on gene promoters, leading to histone deacetylation and gene repression (Doyon *et al.*, 2006; Pena *et al.*, 2006; Shi *et al.*, 2006). Histone deacetylase (HDAC) inhibitors cause the dissociation of the PHD domain containing ING2 subunit from the Sin3 deacetylase complex. The loss of ING2 subsequently disrupts the *in vivo* binding of the Sin3 complex to the p21 promoter (Smith *et al.*, 2010). In this study, we found that *p21* expression was significantly increased in ING2 knockdown embryos. Additionally, there was dramatically decreased expression of *HDAC1* in these ING2 knockdown embryos. These results thus suggest that *p21* as well as *HDAC1* may contribute to the regulation of preimplantation embryonic development. Previous studies show that down-regulation of HDAC1 leads to hyperacetylation of histone H4, which in turn results in developmental delay. The underlying molecular mechanism involves increased expression of *p21*, without any increase in expression of either the pro-apoptotic *Bax* or *Bcl2* transcripts within the HDAC1-depleted embryos (Ma & Schultz, 2008). Additionally, induction of p21 was also observed in Hdac1-deficient embryonic stem cells (Lagger *et al.*, 2002). In summary, this study has demonstrated that ING2 plays important roles in mouse preimplantation development which may be associated with complex epigenetic mechanisms.

Nevertheless, it must be noted that although our results do show that ING2 plays a crucial role in preimplantation development, it is unlikely to be the unique modulator of this process. As seen in Fig. 3*C* and Table 1, only a certain percentage of embryos are affected by microinjection of ING2-specific esiRNA. This could suggest the existence of multiple redundant signalling pathways in preimplantation development. Indeed, it has been hypothesized that this redundancy may provide a fail-safe protection to the preimplantation development programme (Kaye, 1997).

As a basic subunit of the Sin3–HDAC complex, ING2 displays a high degree of interspecies homology between human and mouse (Guerillon *et al.*, 2013). Hence, we hypothesize that ING2 may also play a similar key role in human preimplantation embryonic development. In clinical assisted reproduction, obtaining high-quality embryos is critical for improving the pregnancy rate. Additionally, extended culture and transfer of embryos at the blastocyst stage for assisted reproduction offers some theoretical advantages over the transfer of cleavage-stage embryos. For example, higher implantation rates, single embryo transfer and better temporal synchronization between embryo and endometrium can be achieved by transfer of embryos at the blastocyst stage (Jones *et al.*, 1998; Tsirigotis, 1998; Hambiliki *et al.*, 2013). However, the extended duration of *in vitro* culture involves high risk of reduction in the number of embryos available for transfer, because the *in vitro* culture environment may exert detrimental effects on embryonic development (Jones *et al.*, 1998; Kawamura *et al.*, 2012; Calzi *et al.*, 2012). Hence rigorous characterization of the regulatory mechanisms in embryonic development is of great significance to clinical assisted reproduction. This study sheds some light on the crucial role of ING2 in mouse preimplantation development which gives an inkling on the function of ING2 in human *in vitro* cultured embryos.

### Competing interests

The authors hereby declare that there are no conflicting interests.
